# Does Conceptual Transparency in Manipulatives Afford Place-Value Understanding in Children at Risk for Mathematics Learning Disabilities?

**DOI:** 10.1177/07319487221124088

**Published:** 2022-09-13

**Authors:** Anne Lafay, Helena P. Osana, Joel R. Levin

**Affiliations:** 1 Université Savoie Mont-Blanc, CNRS, LPNC UMR 5105, Chambéry, France; 2 Concordia University, Montreal, Quebec, Canada; 3 The University of Arizona, Tucson, USA

**Keywords:** place value, manipulatives, affordance, mathematics learning disabilities, conceptual transparency

## Abstract

We investigated the effect of conceptual transparency in the physical structure of manipulatives on place-value understanding in typically developing children and those at risk for mathematics learning disabilities. Second graders were randomly assigned to one of three manipulatives conditions: (a) attachable beads that did not make the denominations or ones in the denominations transparent, (b) pipe cleaners that made only the denominations transparent, and (c) string beads that made both the denominations and the ones in the denominations transparent. Participants used the manipulatives to represent double- and triple-digit numerals. Statistical analyses indicated that the transparency of the denominations, but not the transparency of the ones in the denominations, is responsible for children’s number representation and place-value understanding. Descriptive analyses of their responses revealed that the at-risk children were at a greater disadvantage than their typically developing peers with the attachable beads, failing to use place-value concepts to interpret their representations.

Despite its importance, many children struggle to learn the conceptual underpinnings of base-10 number system (Cheung & Ansari, 2020), and low-achieving children exhibit more pronounced difficulties than their typically developing (TD) counterparts (e.g., Chan et al., 2017). Place-value concepts are foundational for elementary mathematics because they allow children to “crack the code” of multidigit numerals and other representations of quantity (Mix et al., 2019). To support children’s understanding of place value, teachers frequently use concrete objects, such as blocks or chips, to represent quantities and perform computations. Providing visual representations with concrete objects, or “manipulatives,” has the potential to highlight place-value concepts and ultimately impact children’s ability to interpret representations of multidigit quantities. Despite being encouraged to use manipulatives; however, teachers are unable to make evidence-based decisions about which types of objects would best support children who have difficulty with place-value concepts. Our objectives in this study were to examine the physical affordances of manipulatives on children’s place-value understanding in the context of representing and interpreting multidigit numerals, and, in particular, to determine the types of objects that are best suited for children who struggle the most in school mathematics.

## Place-Value Knowledge in Children With Mathematics Difficulties

Although the term “place value” has been conceptualized in a number of different ways (e.g., Fuson et al., 1997; Herzog et al., 2019; Ross, 1989), two key principles are at the core of the base-10 number system. First, the position of each digit in a numeral is associated with a specific denomination determined by a power of 10 (e.g., 10^0^, or ones; 10^1^, or tens; 10^2^, or hundreds). Furthermore, the digit itself indicates the number of groups of a specific denomination. In the numeral 26, the 6 represents six groups of 10^0^ (i.e., ones) and the 2 represents two groups of 10^1^ (i.e., tens). Understanding place-value concepts provides meaning to arithmetic (Ho & Cheng, 1997; Moeller et al., 2011) and allows children to draw conceptual connections between different representations of multidigit quantities (Mix et al., 2019).

Children with or at risk for mathematics learning disabilities are those who are low performing and have difficulty learning mathematics, particularly in the areas of counting, enumeration, and basic arithmetic (Dennis et al., 2016). Children with mathematics learning disabilities, or those at risk, also appear to have particular challenges with tasks that rely on with place-value understanding, as evidenced by their difficulties in counting quantities represented with base-ten blocks (Chan et al., 2017), transcoding (Moura et al., 2013), making multidigit magnitude judgments (Landerl & Kölle, 2009), and performing multidigit mental addition (Lambert & Moeller, 2019). Therefore, examining the impact of materials used in educational settings is particularly important for children who have such specific challenges.

## Using Manipulatives in Mathematics

Teachers often use concrete objects to illustrate mathematical concepts, including place value (Mix et al., 2019). Manipulatives are encouraged in part because they have been successfully used with both typical achievers and children with or at risk for mathematics learning disabilities (Satsangi et al., 2016; for reviews, see Carbonneau et al., 2013; Lafay et al., 2019). The benefits of manipulatives have not been demonstrated consistently, however, and the reason could be related to the different physical affordances of the materials used (e.g., plastic cubes, popsicle sticks, and base-10 blocks).

### Conceptual Transparency.

Representations used in the classroom can afford different types of interactions with mathematical concepts (Kaminski & Sloutsky, 2013; McNeil et al., 2009). In the same way, the degree of “conceptual transparency” in manipulatives could impact children’s physical representations of quantities and their interpretations—that is, manipulatives can be effective when they “look like” the concepts they are intended to target, such as illustrating a group of 10 by using a representation that is physically 10 times larger than the representation for a one. We borrow from Chase and Abrahamson (2013) to define the term: A physical object can be considered conceptually transparent when, “the user can access, perceive, and understand its mechanism, logic, and application” (p. 476). Theoretical accounts of conceptual transparency can be drawn from the literature on analogical reasoning (e.g., Gentner & Colhoun, 2010). When manipulatives are used to represent numerals, for example, they can be seen as “pedagogical analogs” (English, 2004) because they share a common underlying structure with place-value concepts. Reasoning with analogies involves a process called “structure mapping” (Gentner, 1983), which entails finding common points between two systems so that a higher order relationship, or schema, can be induced (Gick & Holyoak, 1983; Richland & Simms, 2015).

Drawing out higher-order relationships between two perceptually dissimilar systems does not come naturally to young children, however, who tend to focus on surface similarities (Gentner & Toupin, 1986; Uttal et al., 2008). Because they need instructional support to base their comparisons on structural similarities (Richland et al., 2007; Vendetti et al., 2015), it stands to reason that the more physically similar the two systems are, the greater the likelihood they will abstract the common conceptual structure between them. With respect to manipulatives specifically, proportional manipulatives—those that made the relative sizes of the denominations transparent—were more beneficial for children’s transfer of place-value concepts than objects that relied on color alone to distinguish the size of the denominations (Osana et al., 2018).

### Cognitive Skills Underlying Manipulative Use.

Using manipulatives in base-10 numeration tasks, such as representing quantities, requires several mathematical and cognitive skills. First, counting skill likely plays a central role in place-value tasks that require students to physically represent multidigit quantities, seeing that such tasks would require counting out ones and other denominations. Indeed, as Fuson et al. (1997) and Cobb and Wheatley (1988) argued, counting base-10 units (e.g., tens and ones) is a central mechanism for the development of place-value knowledge. Similarly, we assume that early number sense is required for children to accurately and quickly discriminate between different quantities represented by the objects (e.g., efficiently ascertaining that 9 ones is closer to a group of 10 than 7 ones). Finally, working memory may be required to align elements of concrete and written systems, including in mathematical domains (Mix et al., 2019).

Considerable research has reported that children with or at risk for mathematics learning disabilities differ from their TD peers in place-value understanding (e.g., Chan et al., 2017), counting skill (e.g., Jordan et al., 2007), and early number sense (e.g., Landerl et al., 2009; Mazzocco et al., 2011). Furthermore, there is evidence to suggest that working memory plays an important explanatory role in children’s difficulties in mathematics (e.g., Geary, 2013). Taken together, therefore, it is reasonable to expect that TD children and those with or at risk for mathematics learning disabilities would respond differently to base-10 materials when representing quantities.

## The Current Study

Different varieties of concrete objects have been used to make place-value concepts visible, but no research has explored why some may be more beneficial than others. Objects that “transparently” illustrate place-value concepts are likely to support children’s performance on place-value tasks, but which concepts to make visible to which children is an open question. Furthermore, variability in children’s counting and number sense skills could be reflected in how children work with concrete objects, and any lags in counting and number sense could account for differences in manipulative use between TD children and those with or at risk for mathematics learning disabilities (hereafter referred to as at-risk children).

The objective of the present study was to examine the physical affordances of manipulatives, specifically the transparency of denominations or the number of ones within each denomination, on children’s place-value understanding in the context of constructing and interpreting physical representations of quantity. We also tested whether the manipulatives’ specific features have different effects for at-risk children than for TD children. We asked children to use manipulatives to represent multidigit numerals, and to further probe their place-value understanding, we asked them to verbally interpret their displays. We were interested in examining two physical affordances: the visibility of (a) the denominations, and (b) the ones in the denominations. To this end, we constructed three sets of manipulatives: (a) pipe cleaners cut in lengths that preserved the proportional size of the denominations, making only the denominations visible; (b) beads attached on a string in groups of 10 and 100, making both the denominations and the ones in the denominations visible; and (c) individual beads that could be attached, showing no pre-configured denominations. The two research questions were

**Research Question 1 (RQ1):** Does conceptual transparency in manipulatives support children’s physical representations and interpretations of multidigit numerals?
We expected that the manipulatives that made the denominations transparent would support performance relative to those that did not show the denominations. We also expected that the visibility of the ones in the denominations would support children’s performance compared to manipulatives that hid the exact size of the denominations. Finally, we expected that making both the denominations and the number of ones in the denominations visible would support better performance than materials that made neither transparent.
**Research Question 2 (RQ2):** Does conceptual transparency in manipulatives offer specific advantages in performance on representations and interpretations of multidigit numerals to at-risk children?
For the children in the at-risk group, we expected the same pattern of differences, but with larger effects. In particular, we hypothesized that for at-risk children, who often struggle to interpret the meaning of multidigit numerals, the absence of transparency of the denominations and the number of ones in each denomination would be particularly detrimental.

## Method

### Participants

A total of 123 second-grade children (*M* = 92.0 months, *SD* = 5.2 months; range from 82 to 108 months; 46% female) were recruited from 12 French-speaking public schools in a large urban area in the province of Quebec in Canada. Instruction in all subjects in the participating schools was delivered in French. Table 1 presents the participant demographic information.

**Table 1.** Demographic Information for Second-Grade Study Participants by Mathematics Group.

Variables	Mathematics group			
	TD		At-risk	
	*n*	%	*n*	%
Sex				
Female	43	46.2	13	44.8
Male	50	53.8	16	55.2
SES				
High	23	24.5	6	20.7
Middle	45	47.9	11	37.9
Low	26	27.7	12	41.4
Language				
French	49	53.3	17	60.7
French and English	3	3.3	2	7.1
French, English, and another language	10	10.9	3	10.7
French and another language	30	32.6	6	21.4
Intelligence^a^				
*M* (*SD*)	26.53 (4.61)		20.41 (4.47)	
Range	10–35		11–29	

*Note*. The two groups were equivalent in terms of gender distribution, χ^2^(1, *N* = 122) = .02, *p* = .90. Schools are classified according to a socioeconomic status (SES) index published by Québec’s Ministère de l’Éducation et de l’Enseignement Supérieur (MEES, 2018-2019) based on mother’s education and family income level. All schools in Quebec are ranked from 1 to 10, with lowest indices representing highest SES. We defined high SES as Ranks 1–3, middle as Ranks 4–7, and low as 8–10. The two groups were equivalent in terms of SES distribution, χ^2^(2, *N* = 123) = 1.97, *p* = .37. The two groups were also equivalent in terms of language distribution, χ^2^(3, *N* = 120) = 1.89, *p* = .60. Some children spoke a language other than French and English at home: Arabic 30.6%, Spanish 8.2%, Persian 8.2%, Romanian 8%, Russian 8%, Chinese 6.1%, Kabyle 6.1%, Creole 4.1%, Farsi 4.1%, Mandarin 4.1%, Bosnian 2%, Coreen 2%, Czech 2%, Dari 2%, Gujrati 2%, and Portuguese 2%. TD = typically developing.

a Assessed using Raven’s Colored Progressive Matrices (Raven, 1977).

The sample included TD children and at-risk children. We classified the at-risk children as such because we were not able to use already-existing diagnoses for mathematics learning disabilities to establish our groups (i.e., diagnostic testing is not frequently conducted in the province of Quebec). We used data from teachers and parents and confirmed their reports with an arithmetic fluency measure that we ourselves administered to all children in the sample. Teachers were given a paper-and-pencil survey and asked to choose one of the following options regarding the child: (a) had no mathematics difficulties in class, (b) struggled with mathematics in the class compared to other children, or (c) had a known diagnosis of mathematics learning disabilities. Parents were given the same survey about their child. There were no discrepancies between the responses of the teachers and parents in any case and no child had a known diagnosis of mathematics learning disabilities. No participant was excluded at this point.

We administered the Raven’s Colored Progressive Matrices (Raven, 1977) to assess the participants’ non-verbal intelligence. The reliability estimate for the Raven’s was good, Cronbach’s alpha = .85. Any participant who scored below the 10th percentile would have been removed from the sample, but none fell into this category. We also administered the Tempo Test Rekenen (TTR; De Vos, 1992; Lafay et al., 2020) to all children in the sample. The TTR is a paper-and-pencil test designed to assess arithmetic fluency (i.e., speed and accuracy in solving addition and subtraction problems), a construct that has been used in previous research to determine the presence of mathematics difficulties (e.g., De Visscher & Noël, 2014; Träff et al., 2017). The internal reliability for the TTR was excellent (Cronbach’s α = .92).

We used the TTR and the data from the parents and teachers to classify the children into four groups: those whose parents and teachers reported no mathematics difficulties (94 children), categorized as (a) above the 25th percentile on the TTR (all 94 children, classified as TD) or (b) below the 25th percentile (no child); and those whose parents and teachers reported mathematics difficulties (32 children) who scored (c) above the 25th percentile on the TTR (3 of the 32 children, who were excluded from the sample) and (d) below the 25th percentile (29 students of the 32 children, classified as at-risk). According to Dennis et al. (2016)’s literature review, the 25th percentile was the most frequently used cut-off point for at-risk identification.

The final sample thus consisted of 94 TD children (*M*_age_ = 92.3 months, *SD* = 4.8) and 29 at-risk children (*M*_age_ = 90.7 months, *SD* = 6.3). The two groups did not differ statistically in mean age, *F*(1, 117) = 1.89, *p* = .17. Performance on the TTR was statistically lower in the at-risk sample than in the TD sample, *t*(121) = 5.44, *p* < .001, *d* = 1.16.

### Design and Experimental Manipulation

In each school, children in each of the TD and at-risk groups were randomly assigned in approximately equal numbers to three manipulative conditions: attachable beads (*n* = 39; 30 TD and 9 at-risk), string beads (*n* = 43; 31 TD and 12 at-risk), or pipe cleaners (*n* = 41; 33 TD and 8 at-risk). This resulted in a 2 (Mathematics Group) × 3 (Condition) between-subjects design.

**Figure 1.**
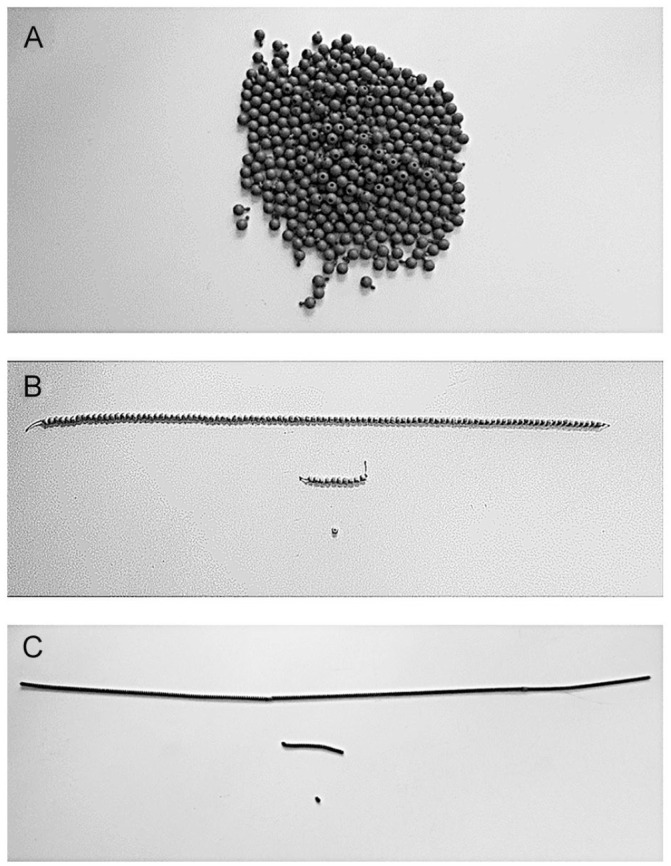
Manipulatives used in each condition. *Note.* Manipulatives used in the three conditions: attachable beads (A), string beads (B), and pipe cleaners (C).

Figure 1 presents the manipulatives used in the three conditions. In the attachable beads condition, children were given a bin with plastic beads that could be attached to form groups. The attachable beads made the ones visible only; no distinct objects were used to represent the denominations. In the string beads condition, children were given wooden beads, some of which were individual and not attachable, and others were strung together in groups of 10 and 100 and were not detachable. The groupings of 10 and 100 beads made the denominations distinct from the ones, and thereby transparent, and the individual beads made the ones in each denomination also visible. In the pipe cleaners condition, children were given a bin with pipe cleaners in proportional lengths: ones were 0.8 cm in length, tens were 10 times as long as the ones (i.e., 8 cm), and hundreds were 10 times as long as the tens (i.e., 80 cm). The pipe cleaners made the denominations transparent only; no ones were visible in the tens or hundreds denominations.

### Measures

#### Cognitive, numeracy, and mathematics abilities

All children first underwent general assessments tapping the cognitive and numerical abilities sensitive to place-value understanding. The assessments were used to ensure no initial condition differences on these measures and to provide a cognitive description of the TD and at-risk groups. We administered two measures of working memory: (a) the Digit Span Backwards (WISC-5, Wechsler, 2014), a standardized test to assess children’s verbal working memory; and (b) the standardized Corsi Block Test (Corsi, 1972) to assess children’s visuospatial working memory. The internal reliability for a composite working memory measure was acceptable (Cronbach’s α = .66). The assessment battery also included two measures of numerical skills: (a) the Numeracy Screener–French version (NS-f, Lafay et al., 2018; originally designed and validated by Nosworthy, 2013), a standardized test that assesses early number sense involving symbolic (Arabic numbers) and non-symbolic (sets of dots) magnitude comparison tasks (Cronbach’s α = .95); and (b) a verbal counting task to assess children’s knowledge of the number sequence and cardinality (Cronbach’s α = .27). The counting task required children to count as high as they could to 30, to skip count by 10 as high as possible to 110, to skip count by 100 as high as possible to 1,100, and to determine how many chips were on the table. The counting score was the number of correct responses out of a maximum of 4 points.

Finally, the Picture Place Value Task (PicPVT), a task developed by the authors (Osana & Lafay, 2019), was administered to assess the children’s understanding of the value of the digits in a numeral. Children were shown a double- or triple-digit numeral with one of the digits underlined. Below the number was presented sets of black hexagons. The number of sets always matched the underlined digit, but the number of hexagons in each set was 1, 10, or 100. Children were required to indicate, by saying “yes” or “no,” whether the picture matched the underlined digit in the numeral. Ten double-digit numerals and 10 triple-digit numerals were randomly ordered, with every child completing the items in the same order. Half of the double-digit items and half of the triple-digit items matched the corresponding picture (e.g., 237 matched the picture of 2 sets of 100 dots). The score was the number of correct responses out of a maximum of 20. The reliability estimate was good (Cronbach’s α = .87).

#### Manipulatives task

##### Task procedure

The manipulatives task consisted of two parts: the representation task and the interpretation task. The researcher began the representation task by showing the manipulatives to the child and describing each denomination. In the attachable beads condition, the researcher constructed a ten by attaching 10 beads together and counting them out loud. She constructed a hundred by attaching 10 tens together in one string, after which she counted out loud to 100 by tens, showing each group of ten between her thumb and index finger. In the string beads and pipe cleaners conditions, the researchers showed the different objects representing the denominations. She counted the number of ones in each denomination, by ones and tens, in the string beads condition. In the pipe cleaners condition, the researcher did not count any ones or tens because these were not visible in the objects. Instead, she used her finger to sweep along each denomination while counting out loud by ones for the tens and by tens for the hundreds denomination. The researcher then demonstrated how to represent 62 with the manipulatives and then counted all 62, first by counting the 60 by tens, and then the two ones, saying “61, 62.”

For the test items, the child was asked to use the manipulatives to represent the numeral indicated on an index card. A legend was left on the table during the entire task, which consisted of a one, a tens denomination, and a hundreds denomination and a laminated index card with the numerals 1, 10, and 100 underneath the corresponding denomination. In the attachable beads condition, the children were provided a bin of single beads. In the other conditions, the children were given three bins of objects, one for each denomination.

Place-value understanding was probed using the interpretation task, on which the children verbally interpreted the meaning of each digit by pointing to its value in their displays. After the children represented the numeral, they were asked to show how each individual digit was represented in their display: “Can you show me this [researcher pointed to each individual digit] in your display?” To interpret their displays, children were permitted to use any of the manipulatives in their display and any in the bins they were provided. The task consisted of two double-digit numerals (i.e., 19 and 32) and two triple-digit numerals (i.e., 127 and 208) to assess the children’s representations of three denominations (i.e., ones, tens, hundreds). No common digits were included in any one numeral to assist us in the interpretation of the children’s responses. The task was video recorded for subsequent data coding.

##### Coding

The total representation score measured the extent to which the child’s representation matched the overall quantity represented by the numeral and the place-value groupings represented by the digits (i.e., groups of tens and hundreds for triple-digit numerals). All responses that were quantitatively accurate and represented the correct number place-value groupings were assigned 1 point. A child received 1 point if he or she represented the numeral 32 with three separate groups of tens and two ones (in the attachable beads condition, the groups of tens needed to be clustered together, either attached or not). All other responses were assigned 0 points. Sample responses receiving 0 points are representing the numeral with 32 individual ones, either attached or not attached; one group of 10 and 22 individual ones; and one set of three ones and one with two ones. The total representation score was the mean number of points received across all four items. Cronbach’s alpha reliability estimate was .66.

We then created five categories to code the types of displays produced on the representation task. The four first categories were constructed using an orthogonal crossing of two dimensions, namely quantitative accuracy and alignment with the place-value groupings in the target numeral. Responses that were quantitatively accurate had the correct number of ones corresponding to the target numeral, regardless of whether the denominational groupings were respected. Responses that were aligned with the place-value groupings showed groupings of tens and ones for double-digit numerals and groupings of hundreds, tens, and ones for three-digit numerals, regardless of whether the number of groups in each denomination corresponded with the target numeral. This resulted in four response categories: (a) Quantitatively accurate and place-value aligned responses (Optimal), (b) Quantitatively accurate without place-value alignment (All-ones), (c) Quantitatively inaccurate with place-value groupings, likely because of counting errors (Grouping), and (d) Quantitatively inaccurate without place-value alignment (Face Value; Barnett-Clarke et al., 2010). The fifth category was Figural responses, characterized by children drawing the numeral with the objects.

We illustrate the categories of responses here with the numeral 32. A response showing three tens and two ones would be coded as optimal because it was quantitatively accurate and matched the place-value groupings in the numeral because the digit 3 was represented with three groups of tens and the digit 2 was represented with two individual ones. An all-ones representation would consist of a set of 32 non-attached ones in any condition or a set of 32 ones attached in one string in the attachable beads condition. A grouping representation could consist of two tens and two ones or four tens and two ones, or any other place-value grouping that resulted in a quantitatively inaccurate display. A face-value response would consist of three ones and two ones. A figural response would be to configure the objects to look like the numeral 32.

A pair of trained research assistants coded 100% of the videos. A second pair independently coded the same set of videos. The videos were approximately evenly distributed between the raters in each pair. This resulted in two sets of independent codes for the whole data set. Percent agreement between the two pairs of coders was 94.4%, which corresponded to a Cohen’s kappa of 0.91. All disagreements were resolved through discussion with the first author.

### Procedure

Testing took place from October to December during the school year. Each child was tested in one individual session lasting 60 min with a researcher. During the session, the researcher administered the mathematics and cognitive measures (i.e., TTR, NS-f, counting task, working memory measures, and non-verbal reasoning task), followed by the PicPVT and the manipulatives task. The administration of the measures was counterbalanced across participants.

### Data Analysis

We first conducted separate 2 (group: TD, at-risk) by 3(condition: attachable beads, string beads, pipe cleaners) analysis of variance (ANOVA) tests on the cognitive and mathematics measures to check for differences between the two mathematics groups as a function of condition. We paid specific attention to the participants’ performance on the PicPVT to provide information on the children’s level of understanding prior to the manipulatives task.

Second, we conducted a 2 (group: TD, at-risk) by 3(condition: attachable beads, string beads, pipe cleaners) ANOVA on the total representation score to test for a group by condition interaction. The omnibus test was followed up with pairwise comparisons to test the specific affordances of interest. In particular, the *denominations visibility* affordance was tested by comparing performance between the pipe cleaners and the attachable beads. The *ones in denominations visibility* affordance was tested by comparing the string beads and the pipe cleaners. Comparing the string beads and the attachable beads served to test the combination of both affordances. Finally, we conducted a response analysis by examining the frequencies of the different types of representations and interpretations observed to reveal patterns of manipulatives use within and across conditions and mathematics groups.

## Results

### Cognitive, Numeracy, and Mathematics Abilities

Both TD and at-risk children had been randomly assigned to the three manipulation conditions and so no between-conditions differences were expected on either of the two numerical measures nor on a composite score of the two working memory measures. Analyses of variance confirmed that expectation. Initial statistical differences between the TD and at-risk children emerged on those measures in all but one of the analyses conducted, with the exception being for working memory in the pipe cleaners condition.

On the PicPVT (range: 0–20), a main effect of group was found, *F*(1, 117) = 32.58, *p* < .001, partial eta-squared = .22, showing that at-risk children (*M* = 10.42; *SD* = 2.61) were less successful than TD children (*M* = 15.94; *SD* = 4.34) on place-value knowledge. To further examine group differences, we created profiles of place-value knowledge by identifying patterns of responses on the correct display and incorrect display items on the PicPVT. Children who correctly answered more than five incorrect-display items and more than five correct-display items were placed in the Proficient-with-Place-Value profile (*n* = 81) as understanding the value of each digit by its position in the numeral. The remaining children were placed in the Lacking-Proficiency-in-Place-Value profile (*n* = 42). Results indicated that a larger proportion of children in the TD group were in the Proficient-with-Place-Value profile (79%) than were the at-risk group (24%), χ^2^(1, *N* = 123) = 29.37, Φ = .49, *p* < .001. Using the more stringent criteria of more than 6 correct in each of the incorrect- and correct-display categories and more than 7 correct in each category yielded similar results. Taken together, these results suggest not only that the at-risk children demonstrated statistically lower performance on the PicPVT than their TD peers, but also that only one quarter of them demonstrated knowledge of the meaning of the digits in multidigit numerals.

### Effects of Manipulatives on Representation Task Performance

Means and standard deviations for the representation task by mathematics group (TD, at-risk) and by condition (attachable beads, string beads, pipe cleaners) are presented in Table 2. To test the effects of the manipulatives and mathematics group on child representation performance, a 2 (group: TD, at-risk) by 3 (condition: attachable beads, string beads, pipe cleaners) ANOVA with unequal cell sizes was performed, based on a Type I error probability of .05 per source of variance. With this analysis, each source of variance (the group main effect, the condition main effect, and the group × condition interaction) was tested while statistically controlling for the effect of the two other sources of variance. Because this was a randomized experimental study with theoretically driven predictions, it was important that the analysis was conducted on equally weighted cell means so that the substantially different cell sizes did not bias the results and conclusions. As a relevant aside, the specific ANOVA procedure that we applied is computationally equivalent to constructing a simultaneous regression model in which the group and condition factors are specially coded variables and the outcome variable of interest (here, representation task performance) is regressed on the coded variables and their products. A main effect of mathematics group was found, *F*(1, 117) = 23.16, *p* < .001, *d* = 1.03, with performance of the at-risk children statistically and substantially lower than that of the TD children.

**Table 2.** Means and (Standard Deviations) on Total Representation Score as a Function of Condition and Mathematics Group.

	TD		At-risk		Unweighted means
Conditions	*n*	*M* (*SD*)	*n*	*M* (*SD*)	
Attachable beads	30	.65 (.34)	9^a^	.37 (.33)	.51
String beads	31	.93 (.17)	12	.63 (.27)	.78
Pipe cleaners	33	.89 (.17)	8	.69 (.35)	.79
Unweighted means		.82		.56	

*Note*. Main entries are the averages of four 0–1 items and therefore range from 0 to 1. The mean square error associated with these data is .0647. TD = typically developing.

a Cell sizes within the at-risk group are unequal because of exclusion criteria and participant absences.

There was also a statistically significant condition effect, *F*(2, 117) = 10.91, *p* < .001, partial eta-squared = .16. Follow-up Fisher Least Significant Difference (LSD) comparisons (Levin et al., 1994) were conducted to test the effects of the two physical affordances. First, to test the effect of the denominations visibility affordance, we compared the mean of the pipe cleaners condition with the mean of the attachable beads condition. The comparison revealed that the children’s performance in the pipe cleaners condition surpassed that in the attachable beads condition, *t*(117) = 4.01, *p* < .001, *d* = 1.10. This finding suggests a benefit of making the denominations visible. Next, to test the effect of visibility of the ones in the denominations, we compared the performance in the string beads condition with that in the pipe cleaners condition. The results revealed no statistical difference between the two conditions, |*t*| < 1, with mean performance in the two conditions essentially equal (see Table 2). This finding suggests no effect of making the ones visible in the denominations.

Finally, to test the effect of visibility of both the denominations and the ones in the denominations relative to the visibility of neither, we compared the performance of the children in the string beads condition with that in the attachable beads condition. A difference emerged between the two conditions, *t*(117) = 4.09, *p* < .001, *d* = 1.05. Superior performance in the string beads condition thus suggests a benefit of making visible both denominations and the ones in the denominations. No group by condition interaction was found, *F* < 1, indicating that condition differences in the children’s mean representation score were statistically comparable for the two mathematics groups and, therefore, this portion of the analysis does not support our initial expectations (see Table 2).

### Response Types and Interpretations by Mathematics Group and Physical Affordance

In this section, we present a description of the children’s responses to gain insight into how the two mathematics groups used the manipulatives in their representations and how they interpreted their displays on the follow-up interpretation task. Our focus was on differences in how the children used the manipulatives as a function of both group (TD and at-risk) and object affordance (i.e., denominations and ones visibility). Thus, the descriptive comparisons reported in this section jointly examine representation types and the children’s follow-up interpretations to obtain a coherent picture of their place-value understanding.

**Figure 2.**
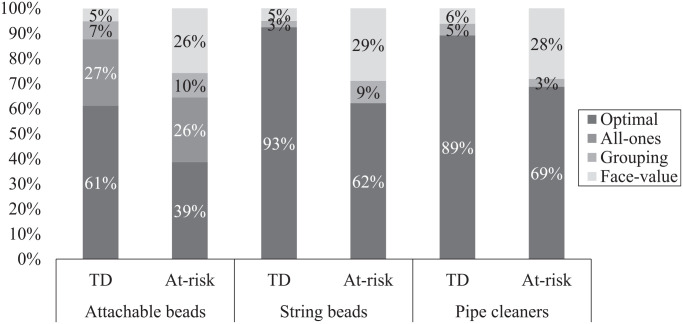
Proportions of response types on the representation task within group and condition. *Note.* Typically developing (TD) children produced 92 responses in the attachable beads condition, 120 responses in the string beads condition, and 130 responses in pipe cleaners condition. Children in the at-risk group produced 29 responses in the attachable beads condition, 45 responses in the string beads condition, and 32 responses in pipe cleaners condition. In the case where we compare groups across conditions, the reported proportions were calculated by dividing the frequency of each response type across the three conditions divided by the total number of responses within each group (i.e., TD or at-risk).

The percentage of responses on the representation task in each mathematics group and in each condition is presented in Figure 2. Some of the percentages reported below are aggregates either across groups or across conditions. The analyses incorporate the children’s responses as the units of analysis because our categorization of responses was specific to the item and not the child, given that each child represented four items with the manipulatives.

#### Mathematics group differences across conditions

In line with the earlier-reported statistical results, the across-conditions percentages on the representation task reveal that at-risk children produced fewer optimal responses than did TD children (TD: 82%; at-risk: 57%). The group differences seem to be explained by the larger percentage of face-value responses in the at-risk group than in the TD group (TD: 5%; at-risk: 28%). These data suggest that the at-risk children had more difficulty than their TD counterparts in understanding the quantitative value of the digits in the target numerals.

#### Effects of physical affordances

With respect to denominations visibility, which was tested by comparing the responses of the children who used attachable beads to those with the pipe cleaners, fewer optimal responses were produced with the attachable beads (56%) than with the pipe cleaners (85%). This discrepancy seems to be explained by the presence of all-ones responses when children used the attachable beads. As shown in Figure 2, all-ones representations (i.e., correctly using ones only) with the attachable beads were produced in both mathematics groups at similar rates (27% and 26% for the TD and at-risk groups, respectively), but such representations were never observed with the pipe cleaners, despite the fact that all-ones responses were possible with those objects.

The percentages presented in Figure 2 further show that the attachable beads were particularly difficult for the children in the at-risk group. Specifically, with the attachable beads, 39% of their responses were optimal compared to 69% with the pipe cleaners and also compared to TD children with either object (61% with attachable beads and 89% with pipe cleaners).

**Figure 3.**
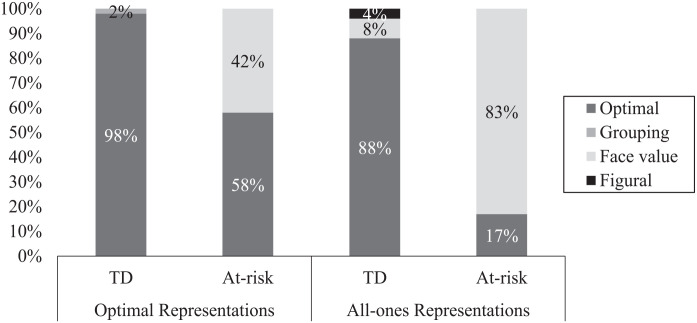
Proportion of interpretation types after optimal and all-ones representations with attachable beads as a function of mathematics group.

Figure 3 presents the percentages of interpretation types in each mathematics group that followed optimal and all-ones representations with attachable beads. These data show additional place-value difficulties for the children with at-risk when using attachable beads. First, 42% of their optimal responses were followed by face-value interpretations. No comparable discrepancy was found when they used the pipe cleaners (82% were followed by optimal interpretations, not shown here) and almost all optimal responses in the TD group were followed by optimal interpretations regardless of object type. Second, 83% of the all-ones representations with the attachable beads in the at-risk group were followed by face-value interpretations, consisting of pointing to individual ones when asked about the tens digit in the target numeral. This stands in contrast to the TD children’s interpretations of their all-ones responses, the vast majority of which (88%) were optimal.

The effects of the ones visibility affordance were examined by comparing the performance of the children in the string beads and pipe cleaners conditions. The proportions in Figure 2 show that across the two mathematics groups, the types of responses on the representation task were distributed similarly in both the string beads and pipe cleaners conditions. Finally, we investigated the effect of combining the two affordances of denominations visibility and ones visibility on children’s performance by comparing the representations in the string beads condition to those in the attachable beads condition. The proportions in Figure 2 reveal similar patterns to those found for the denominations visibility affordance alone (i.e., attachable beads vs. pipe cleaners). The interpretation data also show similar patterns—that is, at-risk children were at a particular disadvantage with the attachable beads, which prompted a relatively larger number of face-value interpretations relative to the string beads.

## Discussion

The objective of the present study was to examine the effects of making place-value concepts transparent in manipulatives on children’s performance on number tasks. In particular, we compared the effects of two different types of conceptual transparency on the performance of second-grade TD and at-risk children. We asked the children to represent multidigit numerals with manipulatives and to explain how the digits in the numerals were represented in their displays. We constructed materials with specific physical features to test two transparency affordances. The first set of materials made the denominations visible, where the groups of tens and hundreds were distinct objects. The denominations were also visible in the second set of materials, but the manipulatives had the added feature of ones visibility—that is, the ones were discrete entities, and therefore countable, in the tens and hundreds objects. To test the physical affordances of denominations visibility and the visibility of the ones in the denominations, we compared these two sets of manipulatives to a third set that consisted of attachable individual ones only, with no distinct objects representing the different denominations and no information about the size of the denominations.

First, we expected that the physical affordances of the manipulatives would systematically affect the children’s performance with the objects and the interpretations of their own displays, regardless of mathematics group membership (TD or at-risk). Performance would be superior when the denominations were visible than when they were not, when the ones in the denominations were visible than when the ones were hidden, and when both the denominations and the ones in the denominations were visible than when neither was visible. Second, we expected an interactive effect between physical affordance and mathematics group. Because of weaker place-value knowledge, number sense, and counting skills of at-risk children, we predicted that those children would be at a greater disadvantage relative to their TD peers when using the attachable beads, manipulatives that made neither of the place-value concepts transparent.

Our predictions regarding the affordances of the manipulatives, regardless of mathematics group, were partially supported. Specifically, performance accuracy was higher when denominations were visible than when they were not and also when both the denominations and ones in the denominations were visible than when neither was visible. At the same time, no difference emerged when the ones in the denominations were visible and when they were not (keeping the visibility of the denominations itself constant). Together, these results suggest that making base-ten groupings transparent affords more accurate physical representations, regardless of the visibility of the ones in those denominations.

The descriptive analysis of the children’s representations indicated that the pipe cleaners and the string beads afforded representations that more frequently displayed place-value groupings than the attachable beads, even if those groupings contained counting errors. In contrast, the attachable beads were the only manipulatives that were conducive to all-ones representations. We suggest that it would be considerably more difficult for children with weak place-value knowledge and counting skills to create their own denominations accurately. An alternate explanation may be that the children were not patient or interested enough to create denominations with the attachable beads, particularly for larger numerals. We rule out this explanation, however, because all the children who produced all-ones representations with the attachable beads connected them one by one into one long string, which also took effort and patience. These observations together allow us to speculate that the visibility of the denominations, by itself, in both the pipe cleaners and the string beads conditions, can account for the stronger performance in these two conditions.

The interactive effects of affordance and mathematics group did not emerge from the statistical analyses, but the descriptive analysis provided insight into why at-risk children struggled relative to the TD children on the representation task. Specifically, the descriptive analysis showed that regardless of object type, the at-risk group produced a proportionally higher number of face-value representations than the TD group, which in part serves to explain the main effect of mathematics group that emerged from the statistical analysis. In addition, we found that all-ones representations were produced at a higher rate with the attachable beads than the other manipulatives, regardless of group. The larger number of all-ones representations served to further disadvantage the at-risk children: Their performance was hampered not only by their mathematics group status (i.e., weaker place-value knowledge, number sense, and counting skill) but also by the physical features of the objects they were given.

We speculate that the at-risk children viewed multidigit quantities as collections of ones rather than as groups of differently-sized units. An illustration of such a “unitary conception of number” (Fuson et al., 1997) is when a child sees the quantity 53 as 53 ones rather than as a quantity decomposed into specific base-10 groupings (i.e., five tens and three ones). With attachable beads, it would be particularly difficult for children with a unitary conception of number to interpret their representations using place-value concepts—even when those representations were optimal. When interpreting the multidigit numerals in the manipulatives task, the at-risk children likely viewed them as “concatenated digits” (Fuson et al., 1997) rather than as symbols that represented base-10 groupings, and there were no physical features in the attachable beads they could use to overcome their unitary conceptions. When they used manipulatives that made the denominations transparent, however, such as the string beads and pipe cleaners, the at-risk children were more likely to provide optimal interpretations of their displays. The TD children, however, were able to marshal their place-value knowledge to better overcome the physical affordances of the manipulatives: They were more likely to correctly interpret their displays regardless of whether the denominations were present in the manipulatives or not.

In contrast to the representation data, the descriptive analyses of the interpretation data suggested an interaction between mathematics group and physical affordance. Specifically, the difference in proportion of face-value interpretations after optimal and all-ones representations in the at-risk group was considerably larger than in the TD group. Thus, the interpretation assessment appeared to be more sensitive to group differences as a function of the physical affordances of manipulatives than the assessment of the children’s representations. We suggest, however, that a more valid and reliable way to access children’s interpretations would be to provide a range of representations that varied on various dimensions (e.g., all-ones, face-value, optimal representations) for children to interpret. Such a refinement to the instrument would allow for more robust quantitative analyses that are nuanced and more useful for practitioners.

### Implications for Practice

Psychologists have made tremendous advances in characterizing the numerical cognition of at-risk children, such as their number processing, magnitude comparison, and transcoding skills (e.g., Lafay et al., 2017; Moura et al., 2013; Rousselle & Noël, 2007). Our research contributes to this literature by documenting the domain-specific knowledge of at-risk children, which has received considerably less research attention. More particularly, the results showed that relative to their TD counterparts, at-risk children produced a larger proportion of face-value representations and were more likely to interpret their all-ones representations, despite being quantitatively accurate, by emphasizing the face values of the digits in the numerals. Although in line with previous work (Chan et al., 2017; Lambert & Moeller, 2019; Landerl & Kölle, 2009; Moura et al., 2013), our study is one of the first to describe the types of place-value difficulties that are closely aligned with the school curriculum and, therefore, particularly valuable in the context of instruction (Newcombe et al., 2009). Given the complex nature of children’s numeration knowledge (Fuson et al., 1997; Herzog et al., 2019), further investigation on other facets of the place-value understanding in this population is warranted.

Furthermore, knowing that conceptual transparency can influence children’s numeration understanding allows educators to tailor their mathematical representations to children’s needs. Our results suggest that making denominations visible by using distinct, and “*unseparatable*,” objects is helpful for children struggling with place value. Despite this finding, however, the design used in the present study did not permit conclusions about children’s *learning* of place value, but only about their performance on number tasks as a function of manipulative type. The implication for teachers is that they should be aware that the materials they choose will influence how children think about the mathematics targeted by those materials. Consistent with the findings of two studies recently published in this journal (Doabler et al., 2020; Liu et al., 2020), our results provide a potentially important instructional consideration: The use of materials will shape children’s thinking about numeration and should be taken into account in the future design of high-quality interventions with at-risk children.

Another key implication of the present findings is related to the degree of information that can be gleaned from place-value tasks typically given in elementary mathematics classrooms. Teachers often ask children to produce representations of multidigit quantities using manipulatives or other materials. Our data suggest that the children’s interpretations of their own representations do not always correspond to what is seen in their displays, and in many instances, expose deep-seated misconceptions that are not evident in their representations. For example, several of the at-risk children displayed place-value groupings when using objects that made the denominations visible, but they could not explain how those groupings corresponded to the digits in the numerals. Conversely, we also observed children correcting mistakes in their representations when they were asked to show the digits in their displays. Our results imply, therefore, that representation tasks alone do not provide teachers with enough information on the quality of their children’s mathematical understandings (Niemi, 1996). As such, when developing and evaluating intervention materials, teachers should consider the ways in which children use and interpret their representations and provide opportunities for discourse related to their use.

### Strengths and Limitations

One of the primary strengths of the present study is that it employed a true experimental design that permitted causal claims about the role of conceptual transparency in children’s mathematics performance. Random assignment to the three experimental conditions diminished the likelihood of initial differences between the conditions, thereby reducing alternative explanations for the findings. Another strength of the study was that we were able to target two specific aspects of place value—the visibility of the denominations and the visibility of the ones in the denominations—by constructing the manipulatives ourselves. Creating manipulatives that were unfamiliar to the participants as mathematics tools also had the effect of controlling for previous exposure to base-10 materials. Aside from likely weak ecological validity resulting from the use of unfamiliar manipulatives, other limitations of the present study include the small sample of at-risk children and the resultant low numbers in each condition in this group.

## Conclusion

The purpose of the present study was to test the influence of conceptual transparency in the physical structure of manipulatives on number representation and numeration understanding in TD and at-risk children. The results suggest that for all children regardless of mathematics group, the physical affordance of transparency did indeed impact their performance. Specifically, the children’s performance was augmented when the denominations were made visible. In addition, fine-grained descriptive analyses of the types of representations the children provided revealed that at-risk children were at a greater disadvantage than their TD peers when the place-value concepts were not visible in the materials. Our study also sheds light on how at-risk children respond to place-value tasks that are typically given in elementary mathematics classrooms: We observed specific difficulties with place value in this population, which up to this point have not been adequately addressed in the literature. A major contribution to the literature is the set of immediately actionable recommendations for educators that stem from the findings.
